# The protective role of mindful parenting against child maltreatment and aggressive behavior: an exploratory study among Chinese parent-adolescent dyads

**DOI:** 10.1186/s13034-022-00507-5

**Published:** 2022-08-30

**Authors:** Lei Yue, Naixue Cui, Nadya Golfenshtein, Naisong Cui, Yinjun Hao, Pingping Lyu

**Affiliations:** 1 School of Nursing and Rehabilitation, Shandong University, No. 44 Wenhuaxi Road, Jinan, 250012 Shandong China; 2 Department of Nursing, Faculty of Social Welfare & Health Sciences, University of Haifa, Haifa, Israel; 3 Rizhao Agricultural College, Rizhao, Shandong China

**Keywords:** Mindful parenting, Adolescent aggressive behavior, Parent-adolescent dyads, Adolescent behavioral health, Child abuse, Parent interaction

## Abstract

**Background:**

It is well-established that child maltreatment practiced by parents is associated with adolescent aggression. Emerging evidence has suggested that higher levels of mindful parenting are associated with fewer negative parenting practices. However, the relationships among mindful parenting, child maltreatment, and adolescent aggression remain unclear.

**Aim:**

To examine the association between mindful parenting, child maltreatment, and adolescent aggressive behavior among Chinese parent-adolescent dyads.

**Methods:**

Survey data from 554 Chinese parent-adolescent dyads were used for the analysis. Parents reported mindful parenting, and adolescents reported three forms of child maltreatment (i.e., physical abuse, psychological aggression, and neglect) by their parents and aggressive behavior. Path models were used to analyze whether higher levels of mindful parenting were associated with decreased likelihood of parent-to-adolescent maltreatment that were further related to lower levels of adolescent aggression.

**Results:**

Mindful parenting and its two factors (i.e., *interaction with full attention* and *compassion and acceptance*) were associated with lower likelihood of physical abuse and psychological aggression, which were related to decreased levels of adolescent aggressive behavior. Stratified analyses by parent gender showed that the path from *interaction with full attention* to adolescent aggression through psychological aggression was also significant or marginally significant in both mother-adolescent and father-adolescent dyads. Stratified analyses by adolescent gender demonstrated that the paths from *interaction with full attention* to physical abuse and psychological aggression were significant, which were also significantly correlated with adolescent aggression among male adolescents, whereas the mindful parenting-child maltreatment-adolescent aggression paths were not significant among female adolescents.

**Conclusions:**

The findings contributed to the existing literature by assessing mindful parenting as a multifaceted construct and exploring the gender differences in the relationships. Gender-tailored interventions to improve mindful parenting, specifically focusing on the parents’ ability of interacting with adolescents providing full attention, compassion, and acceptance may work towards preventing child maltreatment and promoting adolescent behavioral health.

**Supplementary Information:**

The online version contains supplementary material available at 10.1186/s13034-022-00507-5.

## Background

Aggression is one of the most prevalent and destructive behaviors among adolescents [1]. Adolescent aggression is associated with a variety of adjustment problems, such as substance abuse [2], suicide-related behaviors [3], and antisocial behavior disorder [4], which may pose challenges to public health and safety. Cumulative evidence has consistently indicated that child maltreatment is related to adolescent aggressive behavior [5–7]. This may be because child maltreatment practiced by parents is perceived as rejection and hostility by adolescents and elevates their risk of behavioral problems [8]. Alternatively, according to the social learning theory [9], parents endorsing child maltreatment might provide adolescents with a model of aggressive behavior as a normative way to achieve personal goals [10] and solve interpersonal problems [11], increasing adolescents’ aggressive behavior. Therefore, identifying potential parental factors that contribute to child maltreatment can be meaningful to designate tailored interventions to eliminate such negative parenting practices, and in turn prevent adolescent aggression.

Mindful parenting can be an important parental factor that may be protective against child maltreatment. As a construct of growing interest, mindful parenting is defined as the parenting style that involves paying full attention in parent–child interactions, adopting an attitude of non-judgmental acceptance and compassion toward the self as a parent and the child, developing emotional awareness of the self and the child, and exerting self-regulation in the parenting relationship [12, 13]. Mindful parents tend to assume a present-centered and calm attitude and to exhibit greater levels of acceptance and compassion toward their children, which may reduce negative parenting practice, such as harsh and ineffective discipline [14, 15]. A randomized controlled trial indicated that the programs cultivating mindful parenting had the potential to reduce child abuse potential [16].

In line with this, Duncan et al. proposed a theoretical model of mindful parenting’s effects, which depicted that mindful parenting can promote parents to adopt reasonable parenting practices and establish positive parent–child interactions, and in turn, such positive parent–child interactions ultimately decrease negative outcomes for children [12]. Similarly, the Mindful Parenting Effects Model developed by Ahemaitijiang et al. illustrates that mindful parenting can promote parents to adopt proper parenting strategies and form positive family relationship and atmosphere, which can reduce negative outcomes and improve positive outcomes of their children’s development [17]. These models provide solid theoretical justification for exploring the relationship between mindful parenting and adolescent aggression through child maltreatment.

To our best knowledge, two empirical studies tested this relationship empirically. Both studies reported that higher mindful parenting was associated with fewer negative parenting practices (i.e., hostility, physical control, and lax control), which were related to lower levels of internalizing and externalizing symptoms among adolescents [15, 18]. However, both studies collected data only from parents and failed to collect information from children or other informants. It has been suggested that parent–child agreement on child maltreatment [19] and behavioral problems [20] was low. Therefore, it is necessary to further examine these relationships using the data reported by different informants. In addition, previous studies demonstrated that different factors of mindful parenting were differentially associated with parenting practices and child behaviors [21–23]; however, neither study analyzed the specific association of the different factors of mindful parenting with parenting practices and adolescent behaviors.

**Fig. 1:**
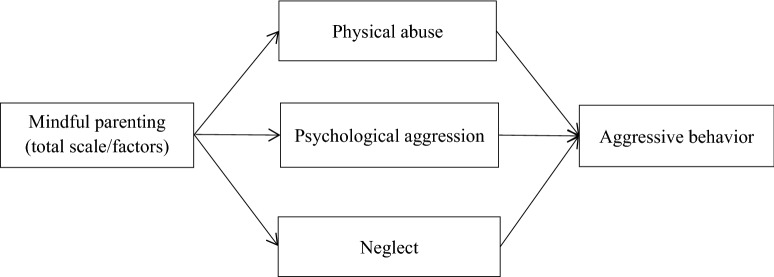
The conceptual framework for the present study.

Based on the existing theoretical models of mindful parenting [12, 17], the present study aimed to examine the protective role of mindful parenting against child maltreatment and adolescent aggression using data collected from parent-adolescent dyads. More specifically, we tested the path model in which mindful parenting and its different factors first related to three forms of child maltreatment (physical abuse, psychological aggression, and neglect) and further to adolescent aggression, as depicted in the conceptual framework in Fig. 1. We hypothesized that higher levels of mindful parenting were associated with a decreased likelihood of child maltreatment, which was related to lower levels of adolescent aggressive behavior.

In addition, considering that mothers and fathers exhibited different mean levels of mindfulness in parenting [24], and that negative maternal and paternal parenting practices had different effects on adolescent behaviors [25, 26], the path models were explored among mother-adolescent dyads and father-adolescent dyads separately in order to better understand whether the protective role of mindful parenting differed between mothers and fathers. Furthermore, given the sex differences in adolescent aggression [27, 28], we also explored the path models in female adolescents and male adolescents separately, in order to understand whether the correlation of mindful parenting with child maltreatment and adolescent aggression differed between female and male adolescents.

## Methods

### Study sample

The study used data from a large survey conducted in 2018, which purposively recruited adolescents from a local vocational secondary school located in a disadvantaged county in eastern China to investigate the relationship between childhood adversities and adolescent mental and behavioral health and its possible psychosocial mechanisms. According to the local and national statistical yearbook of 2018 [29, 30], the percentage of rural population (59.5%) in the study area was much higher than the national percentage (40.4%), and the gross domestic product (GDP) per capita (41,291 CNY; approximately 6,370 USD) was much lower than the national average (70,992 CNY; approximately 10,950 USD). In addition, compared with students at regular high schools, those at the selected vocational school had poor academic performance and usually went directly to the job market after graduation instead of pursuing higher education.

A total of 2904 adolescents were recruited for the large survey study. The parents of the adolescents were invited to participate to provide information on mindful parenting, which was hypothesized as a critical protective factor of childhood adversities, and 649 responded. Among the 649 dyads, 554 dyads provided valid responses on three key variables (i.e., mindful parenting, child maltreatment, and aggressive behavior), and were included in the analysis. Of the 554 dyads, 228 were mother-adolescent and 326 were father-adolescent dyads. A comparison between the adolescents included in the study and those excluded showed that the included adolescents were older (*t* =  − 6.20, *p* < 0.001), more likely to be female (*χ*^*2*^ = 11.68, *p* = 0.001) and only child (*χ*^*2*^ = 5.67, *p* = 0.017), less likely to experience paternal physical abuse (*χ*^*2*^ = 7.50, *p* = 0.006), and exhibit a lower level of aggression (*t* = 2.46, *p* = 0.014, Additional file 1: Table S1). No significant differences in sociodemographic characteristics and mindful parenting were identified between the parents included and excluded in the study, except that the mothers included in this study were more likely to be from rural areas (*χ*^2^ = 7.87, p = 0.020, Additional file 1: Table S2).

### Procedure

We first obtained permission from school officials and head teachers for the research assistants to enter the classrooms. The survey was administered during the self-study period to avoid disruption of regular classes. Informed consent was obtained before distributing the questionnaires to the adolescents. Voluntary participation and withdrawal at any point during the survey were emphasized. After completion of the self-administered questionnaires, the adolescents were asked to bring another questionnaire booklet that consisted of informed consent forms and parent-reported questionnaires to one of their parents after school, and to return the completed questionnaires to the research assistants within a week. The researchers’ contact information was also provided in the questionnaires for parents in case they had any questions. The adolescents were compensated with stationery sets for their participation. A total of 649 parents returned informed consent forms and completed the questionnaires. The study was conducted in accordance with Helsinki Declaration and obtained approval from the Ethics Committee of the School of Nursing of Shandong University (ref no. 2018-R-023).

## Measures

### Aggressive behavior

Adolescents reported their aggressive behavior using the Aggressive Behavior Subscale of the Youth Self Report (YSR, 31). The 17-item Aggressive Behavior Subscale was rated on a 3-point scale (0 = not true, 1 = sometimes true, and 2 = often true). Higher scores indicate more aggressive behavior. The YSR has been validated in Chinese adolescents, showing acceptable to good reliability [32, 33]. In the current study, the Cronbach’s alpha of the subscale was 0.79.

### Mindful parenting

The 24-item Chinese version of the Interpersonal Mindfulness in Parenting Scale (IM-P–C, 34) was used to assess mindful parenting by mothers or fathers. Parent participants rated every item on a five-point scale ranging from 1 (never true) to 5 (always true), and high total scale or subscale scores indicate high levels of mindful parenting. The IM-P–C can be classified into four factors, including *interaction with full attention* (7 items, e.g., “busy thinking while not listening to the child”), *compassion and acceptance* (8 items, e.g., “caring for the child when he/she feels upset”), *self-regulation* (6 items, e.g., “trying to keep the balance of own emotion when upset”), and *emotional awareness of child* (3 items, e.g., “easy to know the child’s feelings”), which were different from the factor structure of the original 31-item IM-P. This was because during the validation process by Pan et al., one item was deleted since it was easily misunderstood with Chinese parents, and six items mainly referring to compassion and acceptance of self were further deleted due to poor corrected item-total correlation and item discriminability [34]. The IM-P–C showed satisfactory to good reliability and good validity among Chinese parents [34]. In the present study, the Cronbach’s alpha was 0.85 for the total scale, 0.61 for the *interaction with full attention* subscale, 0.82 for the *compassion and acceptance* subscale, 0.66 for the *self-regulation* subscale, and 0.38 for the *emotional awareness of child* subscale.

### Child maltreatment

The subscales of psychological aggression (5 items), corporal punishment (5 items), severe physical assault (4 items), very severe physical assault (4 items), and the supplementary subscale of neglect (5 items) in the Chinese version of the Parent–Child Conflict Tactics Scale (CTSPC, 35) were used to assess child maltreatment practiced by fathers and mothers separately in the preceding 12 months on a dichotomized scale (0 = no, 1 = yes) by adolescents. The subscale scores of corporal punishment, severe physical assault, and very severe physical assault were aggregated into one physical abuse score. Sexual abuse was not assessed because sex is a very sensitive topic in Chinese culture and our pilot survey showed that adolescents refused to participate because of items of sexual abuse. Adolescents were regarded as non-physically abused if they rated all items for physical abuse with 0, and otherwise, they were classified as survivors of physical abuse. Similar scoring was applied to identify adolescents with psychological aggression and neglect, respectively. For mother-adolescent dyads, maternal maltreatment was derived, and for father-adolescent dyads, paternal maltreatment was derived. The Chinese version of the CTSPC has been widely applied among youths in China and demonstrated good reliability [19, 36].

### Sociodemographic characteristics

Sociodemographic information, including adolescent age, sex, and only child or not was reported by the participating adolescents. Parents reported their age, relationship to the adolescent (mother vs. father), education (i.e., elementary education or lower, middle school, high school, and college or higher), occupation (i.e., unemployment, unskilled labor, skilled labor, self-employment, and others), family location (i.e., rural area, town, and county or city), and family socioeconomic status (SES). SES was derived from parental education and occupation using the polychoric principal component analysis [37]. Adolescent age, adolescent sex, only child or not, family location, parental relationship to the adolescent, and SES were used as covariates in the path models because they were reported as risk factors for child maltreatment and aggressive behavior [38–41].

### Statistical analysis

First, sample characteristics were summarized using descriptive statistics. Student *t*-tests were then used to examine the differences in adolescent aggressive behavior and mindful parenting between child-maltreated and non-maltreated adolescents, and *Pearson* correlation analysis was used to examine the bivariate association between mindful parenting and aggressive behavior.

Next, following the conceptual framework, parallel mediation path analyses were conducted to examine the path from mindful parenting to aggression through *physical abuse*, *psychological aggression*, and *neglect* adjusting for covariates, considering the co-occurrence of different types of child maltreatment [42]. The parallel mediational path models were first examined among all parent-adolescent dyads, and then repeated in the mother-adolescent dyads, the father-adolescent dyads, parent-female adoelscent dyads, and parent-male adolescent dyads separately. Regarding the analyses of the mother-adolescent dyads, adolescent-reported three forms of maternal maltreatment were used as the mediators, and the same rule was applied to the analyses of the father-adolescent dyads. The above models were run using the total score of mindful parenting and its subscale scores as exogenous variables to explore whether different factors of mindful parenting demonstrated different or similar relationship with child maltreatment and adolescent aggression. Considering the low internal consistency of the mindful parenting subscale *emotional awareness of child*, it was excluded from all analyses.

The significance level was set at *α* = 0.05. Analyses were performed using Stata 15.1 (StataCorp, College Station, Texas, USA).

## Results

### Sample description

Table 1 presents the characteristics of the samples. The included 554 adolescents were aged between 14 and 21 years, with a mean age of 16.91 ± 1.08 years old. There were slightly more males (56.7%) than females (43.3%). Most of the adolescents (89.0%) were not the only child in the family. More fathers (58.8%, mean age 46.37 ± 4.85 years old) participated in the study than mothers (41.2%, mean age 46.00 ± 4.40 years old). The majority of mothers (92.1%) and fathers (84.6%) had an education level of middle school or lower. Most parents had unskilled labor jobs (mothers: 44.7%; fathers: 47.6%) or were self-employed (mothers: 25.9%; fathers: 20.3%). Nearly four-fifths of parents were from rural areas (mothers: 81.1%; fathers: 85.3%). Adolescents in the father-adolescent dyads reported higher levels of SES than those in the mother-adolescent dyads. The most commonly self-reported type of maternal maltreatment was psychological aggression (39.5%), followed by neglect (33.5%) and physical abuse (20.1%), whereas the most commonly self-reported type of paternal maltreatment was neglect (34.9%), followed by psychological aggression (33.1%) and physical abuse (17.2%). The participating parents exhibited a moderate level of mindful parenting, and the participating adolescents engaged in a relatively low level of aggressive behavior.

**Table 1:** Sample characteristics

	Adolescents (*n* = 554)	Mothers (*n* = 228)	Fathers (*n* = 326)
	*n* (%)/M ± SD	*n* (%)/M ± SD	*n* (%)/M ± SD
Age (years)	16.91 ± 1.08	46.00 ± 4.40	46.37 ± 4.85
Sex			
Male	314 (56.7)		
Female	240 (43.3)		
Only child			
No	493 (89.0)		
Yes	61 (11.0)		
Family location			
Rural area		185 (81.1)	278 (85.3)
Town		17 (7.5)	18 (5.5)
County or city		26 (11.4)	30 (9.2)
Parental education			
Elementary or lower		116 (50.9)	65 (19.9)
Middle school		94 (41.2)	211 (64.7)
High school		14 (6.1)	39 (12.0)
College or higher		4 (1.8)	11 (3.4)
Parental occupation			
Unemployment		40 (17.5)	19 (5.8)
Unskilled labor		102 (44.7)	155 (47.6)
Skilled labor		18 (7.9)	64 (19.6)
Self-employment		59 (25.9)	66 (20.3)
Other		9 (4.0)	22 (6.7)
Family SES		− 0.30 ± 1.08	0.20 ± 0.93
Aggressive behavior	7.51 ± 4.40		
Maternal physical abuse			
No	422 (80.4)		
Yes	103 (19.6)		
Paternal physical abuse			
No	435 (82.5)		
Yes	92 (17.5)		
Maternal psychological aggression			
No	358 (65.0)		
Yes	193 (35.0)		
Paternal psychological aggression			
No	378 (68.2)		
Yes	176 (31.8)		
Maternal neglect			
No	360 (68.7)		
Yes	164 (31.3)		
Paternal neglect			
No	342 (65.0)		
Yes	184 (35.0)		
Mindful parenting			
Total scale		3.26 ± 0.55	3.20 ± 0.52
IWFA		3.50 ± 0.49	3.44 ± 0.48
CAA		3.49 ± 0.81	3.43 ± 0.77
SR		2.85 ± 0.78	2.76 ± 0.70
EAC		2.91 ± 0.80	2.93 ± 0.77

*SES* socioeconomic status, *IWFA* interaction with full attention subscale, *CAA* compassion and acceptance subscale, *SR* self regulation subscale, *EAC* emotional awareness of child subscale

### Bivariate analysis results

Compared to non-maltreated counterparts, adolescents who experienced physical abuse (total sample [TS]: *t* = −4.13, *p* < 0.001; mother-adolescent dyads [MA]: *t* =  −3.00, *p* = 0.003; father-adolescent dyads [FA]: *t* = − 2.92, *p* = 0.004) or psychological aggression (TS: *t* = − 4.67, *p* < 0.001; MA: *t* = − 3.19, *p* = 0.002; FA: *t* = − 3.46, *p* < 0.001) reported higher levels of aggressive behavior regardless of perpetrator. Aggression score did not differ significantly among adolescents with and without neglect. Parents perpetrating physical abuse reported lower scores on the total scale of mindful parenting (TS:* t* = 2.49, *p* = 0.013), the *compassion and acceptance* subscale (TS: *t* = 2.74,* p* = 0.006), and the *interaction with full attention* subscale (TS: *t* = 3.78, *p* < 0.001). Parents perpetrating psychological aggression reported lower scores on the *interaction with full attention* subscale (TS: *t* = 2.72, *p* = 0.007) than their counterparts. Neglectful parents did not differ from non-neglectful parents on mindful parenting and its factors.

Analyses among the mother-adolescent dyads showed that physically abusive mothers had lower levels of *interaction with full attention* (*t* = 3.41, *p* < 0.001). Analyses among the father-adolescent dyads revealed that physically abusive fathers exhibited low levels of mindful parenting (*t* = 2.49, *p* = 0.013), *interaction with full attention* (*t* = 2.07, *p* = 0.039), and *compassion and acceptance* (*t* = 2.53, *p* = 0.012), and psychologically aggressive fathers showed lower levels of *interaction with full attention* (*t* = 2.04, *p* = 0.042). See Table 2.

**Table 2:** Bivariate associations of child maltreatment with aggressive behavior and mindful parenting and its factors

	Aggressive behavior		Mindful parenting		Interaction with full attention		Compassion and acceptance		Self regulation	
	M ± SD	*t*	M ± SD	*t*	M ± SD	*t*	M ± SD	*t*	M ± SD	*t*
Total sample										
Physical abuse		− 4.13^***^		2.49^*^		3.78^***^		2.74^**^		0.40
No (427, 80.4%)	7.18 ± 4.05		3.26 ± 0.53		3.50 ± 0.47		3.51 ± 0.78		2.81 ± 0.73	
Yes (104, 19.6%)	9.18 ± 5.42		3.12 ± 0.52		3.31 ± 0.52		3.28 ± 0.76		2.78 ± 0.72	
Psychological aggression		− 4.67^***^		1.37		2.72^**^		1.85		− 0.21
No (356, 64.3%)	6.86 ± 4.07		3.25 ± 0.53		3.51 ± 0.46		3.50 ± 0.78		2.79 ± 0.73	
Yes (198, 35.7%)	8.68 ± 4.72		3.19 ± 0.53		3.39 ± 0.52		3.37 ± 0.78		2.81 ± 0.75	
Neglect		− 1.31		− 0.73		1.15		-1.08		− 0.23
No (348, 65.7%)	7.39 ± 4.38		3.22 ± 0.53		3.48 ± 0.47		3.44 ± 0.80		2.80 ± 0.72	
Yes (182, 34.3%)	7.93 ± 4.48		3.26 ± 0.52		3.43 ± 0.51		3.52 ± 0.75		2.81 ± 0.74	
Mother-adolescent dyads										
Physical abuse		-3.00^**^		0.99		3.41^***^		1.29		− 1.00
No (175, 79.9%)	7.13 ± 3.74		3.28 ± 0.57		3.55 ± 0.46		3.53 ± 0.83		2.83 ± 0.80	
Yes (44, 20.1%)	9.19 ± 4.93		3.19 ± 0.50		3.27 ± 0.56		3.35 ± 0.72		2.96 ± 0.71	
Psychological aggression		− 3.19^**^		0.56		1.94		0.75		0.08
No (138, 60.5%)	6.81 ± 3.93		3.28 ± 0.54		3.55 ± 0.45		3.52 ± 0.80		2.86 ± 0.76	
Yes (90, 39.5%)	8.56 ± 4.07		3.23 ± 0.57		3.42 ± 0.06		3.44 ± 0.83		2.85 ± 0.82	
Neglect		− 1.65		− 1.04		0.32		− 1.46		− 0.28
No (145, 66.5%)	7.22 ± 4.09		3.24 ± 0.59		3.50 ± 0.48		3.44 ± 0.85		2.85 ± 0.79	
Yes (73, 33.5%)	8.20 ± 4.01		3.32 ± 0.48		3.48 ± 0.51		3.61 ± 0.72		2.88 ± 0.77	
Father-adolescent dyads										
Physical abuse		− 2.92^**^		2.49^*^		2.07^*^		2.53^*^		1.56
No (252, 80.8%)	7.21 ± 4.26		3.25 ± 0.50		3.47 ± 0.47		3.50 ± 0.75		2.80 ± 0.68	
Yes (60, 19.2%)	9.17 ± 5.80		3.07 ± 0.53		3.33 ± 0.49		3.22 ± 0.79		2.64 ± 0.70	
Psychological aggression		− 3.46^***^		1.46		2.04^*^		1.90		− 0.23
No (218, 66.9%)	6.89 ± 4.17		3.23 ± 0.53		3.48 ± 0.46		3.49 ± 0.78		2.75 ± 0.71	
Yes (108, 33.1%)	8.78 ± 5.21		3.15 ± 0.49		3.37 ± 0.50		3.32 ± 0.74		2.77 ± 0.69	
Neglect		− 0.41		− 0.07		1.21		− 0.18		− 0.08
No (203, 65.1%)	7.51 ± 4.59		3.21 ± 0.49		3.47 ± 0.46		3.44 ± 0.76		2.76 ± 0.66	
Yes (109, 34.9%)	7.74 ± 4.79		3.22 ± 0.54		3.40 ± 0.51		3.45 ± 0.78		2.77 ± 0.72	

^*^p < 0.05;^**^p < 0.01;^***^p < 0.001

As presented in Table 3, adolescent aggression was significantly and negatively correlated with the *interaction with full attention* subscale score (TS: *r* = −0.13, *p* = 0.002; MA: *r* = − 0.15, *p* = 0.028; FA: *r* = −0.13, *p* = 0.026), whereas its correlations with the total score for mindful parenting and the other two subscale scores were not statistically significant (*r* values from -0.07 to 0.09, *p* values > 0.05).

**Table 3:** Zero-order correlations between aggressive behavior and mindful parenting and its factors

	Total sample	Mother-adolescent dyads	Father-adolescent dyads
	Aggressive behavior	Aggressive behavior	Aggressive behavior
Mindful parenting	− 0.05	− 0.01	− 0.07
Interaction with full attention	− 0.13^**^	− 0.15^*^	− 0.13^*^
Compassion and acceptance	− 0.06	− 0.06	− 0.06
Self regulation	0.03	0.09	− 0.01

^*^p < 0.05;^**^p < 0.01

### Path analysis in the total sample

As shown in Table 4, after controlling for adolescent age, adolescent sex, only child or not, family location, parental relationship to the adolescent, and SES, higher mindful parenting was related to a lower likelihood of physical abuse [*OR* = 0.59; 95% CI = (0.39, 0.89); *p* = 0.012], which was related to low levels of adolescent aggressive behavior (*b* = 1.16, *standard error (se)* = 0.54, *p* = 0.030). Repetitive models using scores of different factors of mindful parenting as the exogenous variable showed that higher *interaction with full attention* subscale score was negatively associated with both psychological aggression [*OR* = 0.60; 95% CI = (0.41, 0.86); *p* = 0.006] and physical abuse [*OR* = 0.41; 95% CI = (0.26, 0.66); *p* < 0.001], which were positively related to lower levels of adolescent aggression (*b* = 1.33, *se* = 0.44, *p* = 0.002; *b* = 1.04, *se* = 0.54, *p* = 0.052, respectively). Additionally, higher *compassion and acceptance* subscale score was related to low levels of adolescent aggression through decreased likelihood of physical abuse [*OR* = 0.68; 95% CI = (0.52, 0.89); *p* = 0.006; *b* = 1.14, *se* = 0.54, *p* = 0.033], and psychological aggression [*OR* = 0.81; 95% CI = (0.65, 1.02); *p* = 0.071; *b* = 1.35, *se* = 0.44, *p* = 0.002). However, the paths from the *self-regulation* subscale of mindful parenting to adolescent aggression through child maltreatment were statistically insignificant.

**Table 4:** The paths from mindful parenting and its different factors to adolescent aggressive behavior through child maltreatment in the total sample

	OR	95% CI	p	b	se	p
MP as the independent variable						
Through physical abuse						
MP → PA	0.59	(0.39,0.89)	0.012			
PA → AB				1.16	0.54	0.030
Through psychological aggression						
MP → PCA	0.80	(0.57,1.11)	0.173			
PCA → AB				1.37	0.44	0.002
Through neglect						
MP → NE	1.16	(0.82,1.65)	0.387			
NE → AB				0.12	0.42	0.768
Direct path from MP to AB						
MP → AB				-0.22	0.36	0.539
IWFA factor as the independent variable						
Through physical abuse						
IWFA → PA	0.41	(0.26,0.66)	< 0.001			
PA → AB				1.04	0.54	0.052
Through psychological aggression						
IWFA → PCA	0.60	(0.41,0.86)	0.006			
PCA → AB				1.33	0.44	0.002
Through neglect						
IWFA → NE	0.82	(0.56,1.19)	0.301			
NE → AB				0.10	0.42	0.815
Direct path from IWFA to AB						
IWFA → AB				-0.86	0.40	0.031
CAA factor as the independent variable						
Through physical abuse						
CAA → PA	0.68	(0.52,0.89)	0.006			
PA → AB				1.14	0.54	0.033
Through psychological aggression						
CAA → PCA	0.81	(0.65,1.02)	0.071			
PCA → AB				1.35	0.44	0.002
Through neglect						
CAA → NE	1.15	(0.91,1.46)	0.233			
NE → AB				0.14	0.42	0.735
Direct path from CAA to AB						
CAA → AB				-0.24	0.24	0.330
SR factor as the independent variable						
Through physical abuse						
SR → PA	0.93	(0.69, 1.26)	0.643			
PA → AB				1.19	0.53	0.026
Through psychological aggression						
SR → PCA	1.02	(0.81,1.29)	0.860			
PCA → AB				1.38	0.44	0.002
Through neglect						
SR → NE	1.04	(0.81,1.34)	0.750			
NE → AB				0.11	0.42	0.796
Direct path from SR to AB						
SR → AB				0.16	0.26	0.539

Path models were adjusted for adolescent age, adolescent sex, only child or not, family location, parental relationship to the adolescent, and family socioeconomic status

*MP* mindful parenting (total scale), *IWFA* interaction with full attention subscale, *CAA* compassion and acceptance subscale, *SR* self regulation subscale, *PA* physical abuse, *PCA* psychological aggression, *NE* neglect, *AB* adolescent aggressive behavior, *OR* odds ratio, *CI* confidence interval, *se* standard error

### Path analysis stratified by parent gender

Among both the mother-adolescent dyads and father-adolescent dyads, the path from *interaction with full attention* subscale score to adolescent aggression through psychological aggression was statistically significant or marginally significant [mother: *OR* = 0.59, 95% CI = (0.34, 1.05), *p* = 0.074; *b* = 1.19, *se* = 0.62, *p* = 0.054; father: *OR* = 0.57, 95% CI = (0.34, 0.95), *p* = 0.030; *b* = 1.48, *se* = 0.60, *p* = 0.013]. In addition, among the father-adolescent dyads, *compassion and acceptance* also related to adolescent aggression through psychological aggression [*OR* = 0.74, 95% CI = (0.55, 1.01), *p* = 0.056; *b* = 1.54, *se* = 0.60, *p* = 0.011]. See Table 5.

**Table 5:** The paths from mindful parenting and its different factors to adolescent aggressive behavior through child maltreatment among mother-adolescent dyads and father-adolescent dyads, respectively

	Mother-adolescent dyads						Father-adolescent dyads					
	*OR*	95% CI	*p*	*b*	*se*	*p*	*OR*	95% CI	*p*	*b*	*se*	*p*
MP as the independent variable												
Through physical abuse												
MP → PA	0.76	(0.42,1.37)	0.357				0.48	(0.27,0.86)	0.013			
PA → AB				1.12	0.77	0.148				1.00	0.73	0.172
Through psychological aggression												
MP → PCA	0.88	(0.54,1.45)	0.622				0.71	(0.45,1.12)	0.137			
PCA → AB				1.19	0.62	0.054				1.55	0.60	0.010
Through neglect												
MP → NE	1.23	(0.72,2.08)	0.454				1.07	(0.67,1.70)	0.775			
NE → AB				0.56	0.61	0.353				-0.20	0.57	0.727
Direct path from MP to AB												
MP → AB				0.13	0.50	0.791				-0.58	0.52	0.260
IWFA factor as the independent variable												
Through physical abuse												
IWFA → PA	0.29	(0.14,0.62)	0.001				0.53	(0.29,0.97)	0.041			
PA → AB				0.92	0.79	0.241				0.97	0.73	0.184
Through psychological aggression												
IWFA → PCA	0.59	(0.34,1.05)	0.074				0.57	(0.34,0.95)	0.030			
PCA → AB				1.19	0.62	0.054				1.48	0.60	0.013
Through neglect												
IWFA → NE	0.82	(0.45,1.48)	0.507				0.77	(0.47,1.27)	0.303			
NE → AB				0.58	0.60	0.337				-0.25	0.56	0.658
Direct path from IWFA to AB												
IWFA → AB				-0.66	0.58	0.259				-1.09	0.54	0.045
CAA factor as the independent variable												
Through physical abuse												
CAA → PA	0.79	(0.52,1.19)	0.266				0.61	(0.42,0.89)	0.010			
PA → AB				1.08	0.77	0.162				1.01	0.73	0.169
Through psychological aggression												
CAA → PCA	0.89	(0.63,1.24)	0.486				0.74	(0.55,1.01)	0.056			
PCA → AB				1.17	0.62	0.059				1.54	0.60	0.011
Through neglect												
CAA → NE	1.23	(0.85,1.79)	0.273				1.07	(0.78,1.46)	0.665			
NE → AB				0.60	0.61	0.325				-0.19	0.57	0.737
Direct path from CAA to AB												
CAA → AB				-0.17	0.34	0.626				-0.36	0.34	0.293
SR factor as the independent variable												
Through physical abuse												
SR → PA	1.23	(0.79,1.91)	0.352				0.71	(0.47,1.09)	0.117			
PA → AB				1.01	0.77	0.193				1.06	0.73	0.148
Through psychological aggression												
SR → PCA	0.99	(0.70,1.40)	0.950				1.05	(0.75,1.46)	0.784			
PCA → AB				1.24	0.62	0.045				1.60	0.60	0.008
Through neglect												
SR → NE	1.04	(0.72,1.50)	0.844				1.03	(0.73,1.45)	0.875			
NE → AB				0.59	0.60	0.332				-0.23	0.57	0.689
Direct path from SR to AB												
SR → AB				0.48	0.35	0.176				-0.07	0.38	0.844

Path models were adjusted for adolescent age, adolescent sex, only child or not, family location, and family socioeconomic status.

*MP* mindful parenting (total scale), *IWFA* interaction with full attention subscale, *CAA* compassion and acceptance subscale, *SR* self regulation subscale, *PA* physical abuse, *PCA* psychological aggression, *NE* neglect, *AB* adolescent aggressive behavior, *OR* odds ratio, *CI* confidence interval, *se* standard error

### Path analysis stratified by adolescent gender

High parental *interaction with full attention* score was related to lower risks of physical abuse [*OR* = 0.44, 95% CI = (0.25, 0.79), *p* = 0.006] and psychological aggression [*OR* = 0.56, 95% CI = (0.34, 0.92), *p* = 0.022] and further lower levels of adolescent aggression (*b* = 1.49, *se* = 0.72, *p* = 0.038; *b* = 1.28, *se* = 0.63, *p* = 0.042, respectively) among male adolescents. The mindful parenting-child maltreatment-adolescent aggression paths were not significant among female adolescents. See Additional file 1: Table S3.

## Discussion

This study revealed that after adjusting adolescent sociodemographic characteristics, mindful parenting, especially its *interaction with full attention* and *compassion and acceptance* factors were negatively associated with physical abuse and psychological aggression, which were positively related to adolescent aggression,. The pathway from higher *interaction with full attention* to lower levels of adolescent aggression through reduced risks of psychological aggression was also evident in both mother-adolescent and father-adolescent dyads. In addition, among father-adolescent dyads, *compassion and acceptance* was related to adolescent aggression through psychological aggression. Stratified analyses by adolescent gender demonstrated that the relationship between *interaction with full attention* and adolescent aggression was mediated by physical abuse and psychological aggression among male adolescents, whereas the mindful parenting-child maltreatment-adolescent aggression paths were not significant among female adolescents. To the best of our knowledge, this is one of the first studies to examine the relationship of mindful parenting and its factors with child maltreatment and adolescent aggression, using data from parent-adolescent dyads.

Although we did not find significant correlation between the total score of mindful parenting and aggression in the zero-order correlation analysis, the *interaction with full attention* factor was significantly and negatively correlated with aggression. Further path analysis also found that after adjusting adolescent sociodemographic covariates, parents with higher levels of mindful parenting, especially *interaction with full attention* and *compassion and acceptance*, were less likely to engaged in physical abuse and psychological aggression towards adolescents. Furthermore, non-maltreated adolescents exhibited low levels of aggressive behavior, which supported the models developed by Duncan et al. [12] and Ahemaitijiang et al. [17], and was consistent with the existing empirical findings [15, 18]. When parents bring their full attention to parent–child interactions, they may perceive their children’s thoughts and feelings more accurately [12], which may reduce parent-adolescent conflicts and disagreements [43]. Furthermore, compassionate parents tend to meet their offspring’s appropriate needs and comfort them when facing negative emotions [12], which facilitates secure parent-adolescent relationship [13]. In addition, parents with acceptance can recognize that parenting challenges, parental limitations, and mistakes are all normal parts of life [44], and, hence, are less likely to provoke conflicts with adolescents. Taken together, parents with higher levels of interaction with full attention and compassion and acceptance are less likely to adopt a reactive posture toward their offspring and are more likely to employ positive parenting practices, which can attenuate adolescent aggressive behavior. In contrast, parents with low levels of interaction with full attention and compassion and acceptance exhibited more punitive parenting practices, such as physical abuse and psychological aggression, which can increase the risks of adolescent aggressive behavior.

Nevertheless, mindful parenting and its different factors were not significantly associated with neglect. In this study, the classification of neglect was driven by the item “showing or telling me that he/she loves me” (mother: 23.6%; father: 25.2%). Culturally, Chinese parents tend to refrain from showing direct affection for their children, and choose to express their affect through providing instrumental support and making sacrifices [45, 46]. Expressing love directly to children is not the focus of mindful parenting, which may explain the insignificant relationship between mindful parenting and neglect. This finding is significant for practical implication as it suggests that mindful parenting interventions may be not a first-line strategy to prevent parental neglect, especially emotional neglect towards adolescents in the Chinese culture.

We did not find a significant relationship between the *self-regulation* factor of mindful parenting and child maltreatment, indicating that not all mindful parenting factors are equally important in reducing child maltreatment. This is, to some extent, consistent with previous findings that different factors of mindful parenting were differentially associated with parenting practices [22, 23]. For example, Rivera et al. found that parents with the skills of nonjudgmental acceptance and acting with awareness were less likely to employ punitive parenting practices, whereas those with the skill of observing were less likely to adopt inconsistent parenting [23]. Our findings suggest that it is important not only to employ the total score of mindful parenting but also to incorporate the individual factor scores to obtain a meaningful understanding of the relationship between mindful parenting and parenting practices, as well as outcomes in children.

Stratified analysis by parent and adolescent gender showed that the protective role of *interaction with full attention* on psychological aggression and adolescent aggression was evident in both mother-adolescent and father-adolescent dyads, while the protective role of *compassion and acceptance* was only evident in the father-adolescent dyads. Furthermore, the paths from *interaction with full attention* to adolescent aggression through physical abuse and psychological aggression were significant in male adolescents, but not in female adolescents. This is inconsistent with the findings from a previous study showing that the protective role of mindful parenting on parenting practices and child externalizing problems was equivalent for mothers and fathers, and for boys and girls [18]. The inconsistency may be due to the smaller sample sizes of the mother-adolescent dyads and female adolescents in our study, which leads to limited statistical power and reduces the chance of detecting true gender differences in the relationships. Nonetheless, given that parenting is considered as a domain with cultural sensitivity [47], cultural differences may be another potential reason for the inconsistent findings. The Western and Chinese parents differed in their understanding of mindful parenting [34], and the relationship between parenting styles and child development showed differential cultural patterns [48]. Taken together, our findings, to some extent, suggest that the gender of child and parent may be taken into consideration in designating mindful parenting interventions. Specifically, fathers may be more sensitive to the *interaction with full attention* and *compassion and acceptance* components of mindful parenting, and male adolescents are more likely to benefit from increasing parental ability of interacting with children with full attention.

The findings should be interpreted cautiously because of limitations. First, the cross-sectional design did not allow the establishment of causal links between the variables. The direction of the association between parenting practices and adolescent behavioral development remained inconsistent, including both reciprocal and unidirectional links [49, 50]. Future studies can test these relationships using longitudinal or experimental designs. Second, we failed to obtain parent-reported data for a large proportion of the adolescent participants, and analyses between included and excluded adolescents showed differences on several variables (e.g., age, sex, only child or not). Although these variables were adjusted in the analytical models, the limited representativeness of the participating parent-adolescent dyads should be acknowledged, and the findings from the study may not be generalizable to the entire population. Future studies using more representative samples of parent-adolescent dyads are needed to replicate the study findings. Third, sexual abuse was not assessed in this study, which can be further investigated. Fourth, the sample sizes of gender-stratified analyses were relatively small, which leads to very limited statistical power and reduces the chance of detecting true gender differences in the relationships. Future studies can recruit larger number of parent-adolescent dyads to further explore the possible gender effect in the relationships among mindful parenting, child maltreatment, and adolescent aggression. Fifth, although several sociodemographic factors were adjusted, other potential factors associated with child maltreatment and adolescent aggressive behavior were not included in this study, such as parental childhood maltreatment history, genetic confounders, and familial factors. Future studies adjusting these covariates are needed to replicate the study findings. Finally, although data from both parents and adolescents were collected, each variable was collected from only one informant. Therefore, the data may have been subject to reporting bias. Future studies are warranted to collect data using a multiple informant approach.

## Conclusions

This study revealed the protective role of mindful parenting and its factors against child maltreatment and adolescent aggressive behavior in a sample of Chinese parent-adolescent dyads. More specifically, high levels of mindful parenting, specifically its *interaction with full attention* and *compassion and acceptance* factors, were associated with decreased likelihood of physical abuse and psychological aggression, and further related to low levels of adolescent aggression. The protective role of *interaction with full attention* against psychological aggression and adolescent aggression was evident in both mother-adolescent and father-adolescent dyads, while the protective role of *compassion and acceptance* was only evident in the father-adolescent dyads. These findings should be interpreted with caution given the unadjusted covariates. Despite this, the findings contributed to the existing literature by assessing mindful parenting as a multifaceted construct and exploring the gender differences in the relationships. In addition, the findings shed light on the importance of gender-tailored interventions on increasing parents’ ability of interacting with adolescents with full attention, compassion, and acceptance, in preventing child maltreatment and promoting adolescent behavioral health.

## Supplementary Information

**Additional file 1: Table S1**. Comparisons between adolescents included in the study and those excluded. **Table S2**. Comparisons between the mothers/fathers included in the study and those excluded. **Table S3. **The paths from mindful parenting and its different factors to adolescent aggressive behavior through child maltreatment among female adolescents and male adolescents, respectively.

## Data Availability

Data and materials are available per reasonable request.

## References

[1] Park S, Chiu W, Won D (2017). Effects of physical education, extracurricular sports activities, and leisure satisfaction on adolescent aggressive behavior: a latent growth modeling approach. PLoS ONE.

[2] Cristello JV, Trucco EM, Zucker RA (2020). Exploring pathways to substance use: a longitudinal examination of adolescent sport involvement, aggression, and peer substance use. Addict Behav.

[3] Hartley CM, Pettit JW, Castellanos D (2018). Reactive aggression and suicide-related behaviors in children and adolescents: a review and preliminary meta-analysis. Suicide Life Threat Behav.

[4] Whipp AM, Korhonen T, Raevuori A, Heikkilä K, Pulkkinen L, Rose RJ (2019). Early adolescent aggression predicts antisocial personality disorder in young adults: a population-based study. Eur Child Adolesc Psychiatry.

[5] Auslander W, Sterzing P, Threlfall J, Gerke D, Edmond T (2016). Childhood abuse and aggression in adolescent girls involved in child welfare: the role of depression and posttraumatic stress. J Child Adolesc Trauma.

[6] Choe C, Yu S (2022). The effect of child abuse and neglect on trajectories of depressive symptoms and aggression in Korean adolescents: exploring gender differences. Int J Environ Res Public Health..

[7] Zhang Y, Ming Q, Wang X, Yao S (2016). The interactive effect of the MAOA-VNTR genotype and childhood abuse on aggressive behaviors in Chinese male adolescents. Psychiatr Genet.

[8] Rohner RP, Khaleque A, Cournoyer DE (2005). Parental acceptance-rejection: theory, methods, cross-cultural evidence, and implications. Ethos.

[9] Bandura A (1978). Social learning theory of aggression. J Commun.

[10] Ross H, Howe N, Rubin KH, Bukowski WM, Laursen B (2009). Family influences on children's peer relationships. Handbook of peer interactions, relationships, and groups.

[11] Wang M (2019). Harsh parenting and adolescent aggression: adolescents' effortful control as the mediator and parental warmth as the moderator. Child Abuse Negl.

[12] Duncan LG, Coatsworth JD, Greenberg MT (2009). A model of mindful parenting: implications for parent-child relationships and prevention research. Clin Child Fam Psychol Rev.

[13] Moreira H, Gouveia MJ, Canavarro MC (2018). Is mindful parenting associated with adolescents’ well-being in early and middle/late adolescence? The mediating role of adolescents' attachment representations, self-compassion and mindfulness. J Youth Adolesc.

[14] Shorey S, Ng ED (2021). The efficacy of mindful parenting interventions: a systematic review and meta-analysis. Int J Nurs Stud.

[15] Parent J, McKee LG, Rough JN, Forehand R (2016). The association of parent mindfulness with parenting and youth psychopathology across three developmental stages. J Abnorm Child Psychol..

[16] Dawe S, Harnett P (2007). Reducing potential for child abuse among methadone-maintained parents: results from a randomized controlled trial. J Subst Abuse Treat.

[17] Ahemaitijiang N, Fang H, Ren Y, Han ZR, Singh NN (2021). A review of mindful parenting: theory, measurement, correlates, and outcomes. J Pac Rim Psychol..

[18] Parent J, Dale C, McKee LG, Sullivan ADW (2021). The longitudinal influence of caregiver dispositional mindful attention on mindful parenting, parenting practices, and youth psychopathology. Mindfulness.

[19] Chan KL (2012). Comparison of parent and child reports on child maltreatment in a representative household sample in Hong Kong. J Fam Violence.

[20] Rey JM, Schrader E, Morris-Yates A (1992). Parent-child agreement on children's behaviours reported by the Child Behaviour Checklist (CBCL). J Adolesc.

[21] Burgdorf V, Szabó M (2020). The interpersonal mindfulness in parenting scale in mothers of children and infants: factor structure and associations with child internalizing problems. Front Psychol.

[22] Potharst ES, Leyland A, Colonnesi C, Veringa IK, Salvadori EA, Jakschik M (2021). Does mothers' self-reported mindful parenting relate to the observed quality of parenting behavior and mother-child interaction?. Mindfulness..

[23] Rivera CE, Coyne LW, Daigle KM, Guzick A, Reid A, Shea S (2022). Mindfulness, parenting behavior, and children's mental health: an investigation among diverse, low-income mothers of preschool aged children. J Contextual Behav Sci.

[24] Medeiros C, Gouveia MJ, Canavarro MC, Moreira H (2016). The indirect effect of the mindful parenting of mothers and fathers on the child's perceived well-being through the child's attachment to parents. Mindfulness.

[25] Cui N, Deatrick JA, Liu J (2018). Maternal and paternal physical abuse: unique and joint associations with child behavioral problems. Child Abuse Negl.

[26] Basili E, Zuffianò A, Pastorelli C, Thartori E, Lunetti C, Favini A (2021). Maternal and paternal psychological control and adolescents' negative adjustment: a dyadic longitudinal study in three countries. PLoS ONE.

[27] Nivette A, Sutherland A, Eisner M, Murray J (2019). Sex differences in adolescent physical aggression: evidence from sixty-three low-and middle-income countries. Aggress Behav.

[28] Archer J (2004). Sex differences in aggression in real-world settings: a meta-analytic review. Rev Gen Psychol.

[29] National Bureau of Statistics (2019). China Statistical Yearbook 2019.

[30] Rizhao Bureau of Statistics (2019). Rizhao Statistical Yearbook 2019.

[31] Achenbach TM (1991). Manual for the Youth Self-Report and 1991 profile.

[32] Cui G, Lan X (2020). The associations of parental harsh discipline, adolescents' gender, and grit profiles with aggressive behavior among Chinese early adolescents. Front Psychol..

[33] Leung PW, Kwong SL, Tang CP, Ho TP, Hung SF, Lee CC (2006). Test-retest reliability and criterion validity of the Chinese version of CBCL, TRF and YSR. J Child Psychol Psychiatry.

[34] Pan J, Liang Y, Zhou H, Wang Y (2019). Mindful parenting assessed in mainland China: psychometric properties of the Chinese version of the Interpersonal Mindfulness in Parenting Scale. Mindfulness.

[35] Straus MA, Hamby SL, Finkelhor D, Moore DW, Runyan D (1998). Identification of child maltreatment with the parent-child conflict tactics scales: development and psychometric data for a national sample of American parents. Child Abuse Negl.

[36] Cui NX, Xue J, Connolly CA, Liu JH (2016). Does the gender of parent or child matter in child maltreatment in China?. Child Abuse Negl.

[37] Mtei G, Borghi J, Hanson K (2015). Predicting consumption expenditure for the analysis of health care financing equity in low income countries: a comparison of approaches. Soc Indic Res.

[38] Christian CW (2015). The evaluation of suspected child physical abuse. Pediatrics.

[39] Liao M, Lee AS, Roberts-Lewis AC, Hong JS, Jiao K (2011). Child maltreatment in China: an ecological review of the literature. Child Youth Serv Rev.

[40] Liu J (2011). Early health risk factors for violence: conceptualization, review of the evidence, and implications. Aggress Violent Behav.

[41] World Health Organization. https://www.who.int/publications/i/item/preventing-child-maltreatment-a-guide-to-taking-action-and-generating-evidence (2006). Accessed 15 Aug 2021.

[42] Kim K, Mennen FE, Trickett PK (2017). Patterns and correlates of co-occurrence among multiple types of child maltreatment. Child Fam Soc Work.

[43] Park YR, Nix RL, Duncan LG, Coatsworth JD, Greenberg MT (2020). Unfolding relations among mindful parenting, recurrent conflict, and adolescents' externalizing and internalizing problems. Fam Process.

[44] Moreira H, Cristina CM (2020). Mindful parenting is associated with adolescents' difficulties in emotion regulation through adolescents' psychological inflexibility and self-compassion. J Youth Adolesc.

[45] Chao R, Tseng V, Bornstein MH (2002). Parenting of Asians. Handbook of parenting. Social conditions and applied parenting.

[46] Cheah CS, Li J, Zhou N, Yamamoto Y, Leung CY (2015). Understanding Chinese immigrant and European American mothers' expressions of warmth. Dev Psychol.

[47] Sigel IE, McGillicuddy-DeLisi AV, Goodnow JJ (2014). Parental belief systems: The psychological consequences for children.

[48] Porter C, Hart C, Yang C, Robinson C, Frost Olsen S, Zeng Q (2005). A comparative study of child temperament and parenting in Beijing, China and the western United States. Int J Behav Dev.

[49] Wang M, Liu L (2018). Reciprocal relations between harsh discipline and children's externalizing behavior in China: a 5-year longitudinal study. Child Dev.

[50] Lansford JE, Criss MM, Laird RD, Shaw DS, Pettit GS, Bates JE (2011). Reciprocal relations between parents' physical discipline and children's externalizing behavior during middle childhood and adolescence. Dev Psychopathol.

