# Supplementary material for: The protective role of mindful parenting against child maltreatment and aggressive behavior: an exploratory study among Chinese parent-adolescent dyads
Source: Child Adolesc Psychiatry Ment Health. 2022 Aug 30;16:72. doi: 10.1186/s13034-022-00507-5 (PMC9429749; doi:10.1186/s13034-022-00507-5)
Supplement: Supplementary file 1 — Additional file 1: Table S1. Comparisons between adolescents included in the study and those excluded. Table S2. Comparisons between the mothers/fathers included in the study and those excluded. Table S3. The paths from mindful parenting and its different factors to adolescent aggressive behavior through child maltreatment among female adolescents and male adolescents, respectively. [file 13034_2022_507_MOESM1_ESM.docx]

**Supplementary Table S1**. Comparisons between adolescents included in the study and those excluded.

|  | Included adolescents (*n* = 554) |  | Excluded adolescents |  |
| --- | --- | --- | --- | --- |
|  | *n* (%)/M ± SD |  | *n* (%)/M ± SD | χ^2^/t |
| Age (years)（*n_e_* = 2073) | 16.91 ± 1.08 |  | 16.63 ± 0.92 | -6.20^***^ |
| Sex (*n_e_* = 2116) |  |  |  | 11.68^**^ |
| Male | 314 (56.7) |  | 1366 (64.6) |  |
| Female | 240 (43.3) |  | 750 (35.4) |  |
| Only child (*n_e_* = 1812) |  |  |  | 5.67^*^ |
| No | 493 (89.0) |  | 1671 (92.2) |  |
| Yes | 61 (11.0) |  | 141 (7.8) |  |
| Maternal physical abuse (*n_e_* = 1974) |  |  |  | 0.43 |
| No | 422 (80.4) |  | 1561 (79.1) |  |
| Yes | 103 (19.6) |  | 413 (20.9) |  |
| Paternal physical abuse (*n_e_* = 1978) |  |  |  | 7.50^**^ |
| No | 435 (82.5) |  | 1523 (77.0) |  |
| Yes | 92 (17.5) |  | 455 (23.0) |  |
| Maternal psychological aggression (*n_e_* = 2121) |  |  |  | 0.416 |
| No | 358 (65.0) |  | 1417 (66.8) |  |
| Yes | 193 (35.0) |  | 704 (33.2) |  |
| Paternal psychological aggression (*n_e_* = 2121) |  |  |  | 0.460 |
| No | 378 (68.2) |  | 1412 (66.6) |  |
| Yes | 176 (31.8) |  | 709 (33.4) |  |
| Maternal neglect (*n_e_* = 1966) |  |  |  | 0.748 |
| No | 360 (68.7) |  | 1365 (69.4) |  |
| Yes | 164 (31.3) |  | 601 (30.6) |  |
| Paternal neglect (*n_e_* = 1977) |  |  |  | 0.375 |
| No | 342 (65.0) |  | 1326 (67.1) |  |
| Yes | 184 (35.0) |  | 651 (32.9) |  |
| Aggressive behavior (*n_e_* = 2023) | 7.51 ± 4.40 |  | 8.11 ± 5.17 | 2.46^*^ |

*Notes.* *n_e_*, sample size of adolescents who were excluded from the study but with available data on the variables. *^*^p*＜0.05;*^**^p*＜0.01;*^***^p*＜0.001

**Supplementary Table S2**. Comparisons between the mothers/fathers included in the study and those excluded.

|  | Included parents  M (*n =* 228)/F (*n =* 326) |  | Excluded parents  M (*n =* 35)/F (*n =* 58) |  |
| --- | --- | --- | --- | --- |
|  | *n* (%)/M ± SD |  | *n* (%)/M ± SD | χ^2^/t |
| Age (years) |  |  |  |  |
| Female | 46.00 ± 4.40 |  | 44.75 ± 3.43 | -1.60 |
| Male | 46.37 ± 4.85 |  | 45.07 ± 5.05 | -1.88 |
| Sex |  |  |  | 0.41 |
| Female | 228 (41.2) |  | 35 (37.6) |  |
| Male | 326 (58.8) |  | 58 (62.4) |  |
| Maternal family location |  |  |  | 7.87^*^ |
| Rural area | 185 (81.1) |  | 22 (62.9) |  |
| Town | 17 (7.5) |  | 3 (8.6) |  |
| County or city | 26 (11.4) |  | 10 (28.5) |  |
| Paternal family location |  |  |  | 1.55 |
| Rural area | 278 (85.3) |  | 53 (91.3) |  |
| Town | 18 (5.5) |  | 2 (3.5) |  |
| County or city | 30 (9.2) |  | 3 (5.2) |  |
| Maternal education |  |  |  | 3.57 |
| Elementary or lower | 116 (50.9) |  | 21 (60.0) |  |
| Middle school | 94 (41.2) |  | 10 (28.6) |  |
| High school | 14 (6.1) |  | 4 (11.4) |  |
| College or higher | 4 (1.8) |  | 0 (0.0) |  |
| Paternal education |  |  |  | 3.64 |
| Elementary or lower | 65 (19.9) |  | 14 (24.1) |  |
| Middle school | 211 (64.7) |  | 40 (69.0) |  |
| High school | 39 (12.0) |  | 4 (6.9) |  |
| College or higher | 11 (3.4) |  | 0 (0.0) |  |
| Maternal occupation |  |  |  | 8.17 |
| Unemployment | 40 (17.5) |  | 7 (20.0) |  |
| Unskilled labor | 102 (44.7) |  | 10 (28.6) |  |
| Skilled labor | 18 (7.9) |  | 7 (20.0) |  |
| Self-employment | 59 (25.9) |  | 8 (22.9) |  |
| Other | 9 (4.0) |  | 3 (8.5) |  |
| Paternal occupation |  |  |  | 1.93 |
| Unemployment | 19 (5.8) |  | 2 (3.5) |  |
| Unskilled labor | 155 (47.6) |  | 30 (51.7) |  |
| Skilled labor | 64 (19.6) |  | 14 (24.1) |  |
| Self-employment | 66 (20.3) |  | 9 (15.5) |  |
| Other | 22 (6.7) |  | 3 (5.2) |  |
| Maternal family SES | -0.30 ± 1.08 |  | -0.25 ± 1.12 | -0.24 |
| Paternal family SES | 0.20 ± 0.93 |  | 0.12 ± 0.78 | 0.67 |
| Maternal mindful parenting |  |  |  |  |
| Total scale | 3.26 ± 0.55 |  | 3.29 ± 0.54 | 0.25 |
| IWFA | 3.50 ± 0.49 |  | 3.57 ± 0.53 | 0.79 |
| CAA | 3.49 ± 0.81 |  | 3.53 ± 0.87 | 0.28 |
| SR | 2.85 ± 0.78 |  | 2.73 ± 0.80 | -0.90 |
| EAC | 2.91 ± 0.80 |  | 3.09 ± 0.79 | 1.27 |
| Paternal mindful parenting |  |  |  |  |
| Total scale | 3.20 ± 0.52 |  | 3.24 ± 0.55 | 0.52 |
| IWFA | 3.44 ± 0.48 |  | 3.52 ± 0.52 | 1.08 |
| CAA | 3.43 ± 0.77 |  | 3.45 ± 0.80 | 0.17 |
| SR | 2.76 ± 0.70 |  | 2.81 ± 0.74 | 0.47 |
| EAC | 2.93 ± 0.77 |  | 2.92 ± 0.80 | -0.08 |

*Note.* M, mothers. F, fathers. SES, socioeconomic status. IWFA, interaction with full attention subscale. CAA, compassion and acceptance subscale. SR, self regulation subscale. EAC, emotional awareness of child subscale. *^*^p*＜0.05

**Supplementary Table S3.** The paths from mindful parenting and its different factors to adolescent aggressive behavior through child maltreatment among female adolescents and male adolescents, respectively.

|  | Female adolescents | | | | | | |  | Male adolescents | | | | | | |
| --- | --- | --- | --- | --- | --- | --- | --- | --- | --- | --- | --- | --- | --- | --- | --- |
|  | *OR* | 95% CI | *p* |  | *b* | *se* | *p* |  | *OR* | 95% CI | *p* |  | *b* | *se* | *p* |
| **MP as the independent variable** |  |  |  |  |  |  |  |  |  |  |  |  |  |  |  |
| *Through physical abuse* |  |  |  |  |  |  |  |  |  |  |  |  |  |  |  |
| MP *→* PA | 0.45 | (0.21,0.96) | 0.040 |  |  |  |  |  | 0.70 | (0.43,1.16) | 0.171 |  |  |  |  |
| PA *→* AB |  |  |  |  | 0.66 | 0.81 | 0.411 |  |  |  |  |  | 1.60 | 0.72 | 0.026 |
| *Through psychological aggression* |  |  |  |  |  |  |  |  |  |  |  |  |  |  |  |
| MP *→* PCA | 0.78 | (0.46,1.33) | 0.369 |  |  |  |  |  | 0.78 | (0.51,1.21) | 0.268 |  |  |  |  |
| PCA *→* AB |  |  |  |  | 1.47 | 0.59 | 0.013 |  |  |  |  |  | 1.32 | 0.63 | 0.036 |
| *Through neglect* |  |  |  |  |  |  |  |  |  |  |  |  |  |  |  |
| MP *→* NE | 1.03 | (0.60,1.77) | 0.905 |  |  |  |  |  | 1.22 | (0.77,1.95) | 0.402 |  |  |  |  |
| NE *→* AB |  |  |  |  | 0.28 | 0.56 | 0.622 |  |  |  |  |  | 0.05 | 0.60 | 0.934 |
| *Direct path from MP to AB* |  |  |  |  |  |  |  |  |  |  |  |  |  |  |  |
| MP *→* AB |  |  |  |  | 0.25 | 0.50 | 0.624 |  |  |  |  |  | -0.50 | 0.51 | 0.323 |
| **IWFA factor as the independent variable** |  |  |  |  |  |  |  |  |  |  |  |  |  |  |  |
| *Through physical abuse* |  |  |  |  |  |  |  |  |  |  |  |  |  |  |  |
| IWFA *→* PA | 0.30 | (0.13,0.73) | 0.008 |  |  |  |  |  | 0.44 | (0.25,0.79) | 0.006 |  |  |  |  |
| PA *→* AB |  |  |  |  | 0.45 | 0.81 | 0.573 |  |  |  |  |  | 1.49 | 0.72 | 0.038 |
| *Through psychological aggression* |  |  |  |  |  |  |  |  |  |  |  |  |  |  |  |
| IWFA *→* PCA | 0.65 | (0.37,1.15) | 0.143 |  |  |  |  |  | 0.56 | (0.34,0.92) | 0.022 |  |  |  |  |
| PCA *→* AB |  |  |  |  | 1.44 | 0.59 | 0.014 |  |  |  |  |  | 1.28 | 0.63 | 0.042 |
| *Through neglect* |  |  |  |  |  |  |  |  |  |  |  |  |  |  |  |
| IWFA *→* NE | 0.70 | (0.40,1.25) | 0.229 |  |  |  |  |  | 0.92 | (0.56,1.54) | 0.763 |  |  |  |  |
| NE *→* AB |  |  |  |  | 0.24 | 0.56 | 0.665 |  |  |  |  |  | 0.03 | 0.60 | 0.958 |
| *Direct path from IWFA to AB* |  |  |  |  |  |  |  |  |  |  |  |  |  |  |  |
| IWFA *→* AB |  |  |  |  | -0.70 | 0.54 | 0.194 |  |  |  |  |  | -0.99 | 0.56 | 0.077 |
| **CAA factor as the independent variable** |  |  |  |  |  |  |  |  |  |  |  |  |  |  |  |
| *Through physical abuse* |  |  |  |  |  |  |  |  |  |  |  |  |  |  |  |
| CAA *→* PA | 0.56 | (0.34,0.93) | 0.026 |  |  |  |  |  | 0.76 | (0.54,1.07) | 0.111 |  |  |  |  |
| PA *→* AB |  |  |  |  | 0.64 | 0.81 | 0.432 |  |  |  |  |  | 1.58 | 0.72 | 0.028 |
| *Through psychological aggression* |  |  |  |  |  |  |  |  |  |  |  |  |  |  |  |
| CAA *→* PCA | 0.77 | (0.54,1.09) | 0.145 |  |  |  |  |  | 0.83 | (0.62,1.11) | 0.212 |  |  |  |  |
| PCA *→* AB |  |  |  |  | 1.47 | 0.59 | 0.013 |  |  |  |  |  | 1.32 | 0.63 | 0.036 |
| *Through neglect* |  |  |  |  |  |  |  |  |  |  |  |  |  |  |  |
| CAA *→* NE | 1.12 | (0.78,1.60) | 0.548 |  |  |  |  |  | 1.15 | (0.84,1.59) | 0.381 |  |  |  |  |
| NE *→* AB |  |  |  |  | 0.28 | 0.57 | 0.622 |  |  |  |  |  | 0.07 | 0.60 | 0.913 |
| *Direct path from CAA to AB* |  |  |  |  |  |  |  |  |  |  |  |  |  |  |  |
| CAA *→* AB |  |  |  |  | 0.07 | 0.34 | 0.832 |  |  |  |  |  | -0.42 | 0.35 | 0.221 |
| **SR factor as the independent variable** |  |  |  |  |  |  |  |  |  |  |  |  |  |  |  |
| *Through physical abuse* |  |  |  |  |  |  |  |  |  |  |  |  |  |  |  |
| SR *→* PA | 0.96 | (0.56,1.62) | 0.868 |  |  |  |  |  | 0.99 | (0.68,1.44) | 0.950 |  |  |  |  |
| PA *→* AB |  |  |  |  | 0.63 | 0.80 | 0.432 |  |  |  |  |  | 1.64 | 0.72 | 0.022 |
| *Through psychological aggression* |  |  |  |  |  |  |  |  |  |  |  |  |  |  |  |
| SR *→* PCA | 1.04 | (0.72,1.50) | 0.845 |  |  |  |  |  | 1.00 | (0.73,1.37) | 0.980 |  |  |  |  |
| PCA *→* AB |  |  |  |  | 1.45 | 0.59 | 0.014 |  |  |  |  |  | 1.35 | 0.63 | 0.031 |
| *Through neglect* |  |  |  |  |  |  |  |  |  |  |  |  |  |  |  |
| SR *→* NE | 0.98 | (0.67,1.43) | 0.930 |  |  |  |  |  | 1.04 | (0.73,1.46) | 0.844 |  |  |  |  |
| NE *→* AB |  |  |  |  | 0.29 | 0.56 | 0.606 |  |  |  |  |  | 0.01 | 0.60 | 0.992 |
| *Direct path from SR to AB* |  |  |  |  |  |  |  |  |  |  |  |  |  |  |  |
| SR *→* AB |  |  |  |  | 0.41 | 0.35 | 0.239 |  |  |  |  |  | 0.04 | 0.38 | 0.910 |

*Note.* Path models were adjusted for adolescent age, only child or not, family location, parental relationship to the adolescent, and family socioeconomic status. MP, mindful parenting (total scale). IWFA, interaction with full attention subscale. CAA, compassion and acceptance subscale. SR, self regulation subscale. PA, physical abuse. PCA, psychological aggression. NE, neglect. AB, adolescent aggressive behavior. OR, odds ratio. CI, confidence interval. se, standard error.
